# How to Preserve Steatotic Liver Grafts for Transplantation

**DOI:** 10.3390/jcm12123982

**Published:** 2023-06-12

**Authors:** Damiano Patrono, Nicola De Stefano, Elena Vissio, Ana Lavinia Apostu, Nicoletta Petronio, Giovanni Vitelli, Giorgia Catalano, Giorgia Rizza, Silvia Catalano, Fabio Colli, Luigi Chiusa, Renato Romagnoli

**Affiliations:** 1 General Surgery 2U—Liver Transplant Unit, Department of Surgical Sciences, Azienda Ospedaliero Universitaria Città della Salute e della Scienza di Torino, Università di Torino, Corso Bramante 88–90, 10126 Turin, Italy; 2 Department of Pathology, Azienda Ospedaliero Universitaria Città della Salute e della Scienza di Torino, Università di Torino, Corso Bramante 88–90, 10126 Turin, Italy

**Keywords:** macrovesicular steatosis, ischemia-reperfusion injury, preservation solution, polyethylene glycol, ischemic preconditioning, hypothermic oxygenated machine perfusion, normothermic machine perfusion, ischemia-free liver transplantation

## Abstract

Liver allograft steatosis is a significant risk factor for postoperative graft dysfunction and has been associated with inferior patient and graft survival, particularly in the case of moderate or severe macrovesicular steatosis. In recent years, the increasing incidence of obesity and fatty liver disease in the population has led to a higher proportion of steatotic liver grafts being used for transplantation, making the optimization of their preservation an urgent necessity. This review discusses the mechanisms behind the increased susceptibility of fatty livers to ischemia-reperfusion injury and provides an overview of the available strategies to improve their utilization for transplantation, with a focus on preclinical and clinical evidence supporting donor interventions, novel preservation solutions, and machine perfusion techniques.

## 1. Introduction

Since the early days of liver transplantation (LT), liver allograft steatosis has emerged as a major risk factor for graft dysfunction and it has been associated with inferior patient and graft survival [1]. In past years, the histological definition and quantification of steatosis has been widely heterogeneous [2,3,4], which is reflected in the striking variability in the assessment of its impact on LT outcomes [5,6,7,8]. Steatosis has been most frequently distinguished as macrovesicular steatosis (MaS, or large droplet fat) and microvesicular steatosis (small/medium droplet fat). MaS is characterized by the presence of a single large fat vacuole displacing the nucleus towards the periphery of the hepatocytes and distending cell membrane to a larger size compared to surrounding non-steatotic hepatocytes [9], whereas small/medium droplet fat is identified as smaller fat vacuoles not meeting the above definition for MaS. 

In LT, the clinical implications of the two types of hepatic steatosis are very different. Although the presence of ≥30% microvesicular steatosis has been associated with an increased risk of postreperfusion syndrome, early allograft dysfunction [10], rejection, and the need for postoperative renal replacement therapy [11,12], use of liver grafts with even significant microvesicular steatosis is generally considered to be safe [9,13]. In contrast, the presence of moderate (30–60%) or severe (≥60%) MaS appears to be more clinically impactful, proportionally to its severity. The utilization of livers with moderate MaS has been associated with an increased rate of early allograft dysfunction, biliary complications, and decreased graft survival, whereas severe MaS has been linked to postoperative poor function, need for renal replacement therapy, and inferior patient and graft survival [5,6]. Consequently, MaS has been included as a negative prognostic factor in models predicting post-LT patient and graft survival [14,15]. The high risk associated with the use of severely steatotic livers is reflected by the very low number of patients in the series reporting their use [7,16,17,18,19], suggesting that, despite some encouraging results that have been reported, these grafts are generally approached with extreme caution and most frequently discarded. Recently, a study based on the Scientific Registry of Transplant Recipients has shown that moderate (≥31%) liver allograft MaS is associated with 87% to 95% lower odds of graft utilization, while utilization of fatty livers increases the risk of graft failure by 53% [20].

Hepatic steatosis is expected to become more frequent among organ donors, as the prevalence of overweight and obesity in the world adult population has been estimated to be 39% and 13%, respectively [21]. In the United States, projections show that by 2030, 48.9% of adult population will be obese (BMI ≥ 30) and 24.2% will be severely obese (BMI ≥ 35) [22]. Consequently, the global prevalence of non-alcoholic or metabolic-associated fatty liver disease, of which hepatic steatosis represents the distinctive feature, has been estimated to be about 25% [23,24]. 

The increasing incidence of obesity and fatty liver disease has obvious consequences on LT activity, with non-alcoholic steatohepatitis (NASH) representing the fastest rising indication for LT in many countries [25,26]. Furthermore, between 2002 and 2016, the prevalence of NASH-related HCC and HCC in LT candidates with NASH increased 7.7-fold and 11.8-fold, respectively, in the United States [27]. 

Thus, given the increasing incidence of hepatic steatosis in the population and the detrimental impact of moderate or severe MaS on LT outcomes, it appears that simply discarding steatotic grafts does not represent a viable option. As the safe use of steatotic grafts may help relieve the chronic organ shortage experienced by most transplant organizations, strategies to optimize fatty liver preservation and improve LT outcomes are urgently needed. 

In the past, transplant surgeons have strived to improve the outcomes of LT using fatty livers by minimizing additional risk factors, such as avoiding their use in recipients with severe hepatopathy or limiting cold ischemia time [18,28,29]. In recent years, however, several interventions have been proposed to reduce preservation injury in fatty livers, including donor interventions, use of novel preservation solutions, and machine perfusion techniques. After briefly recapping the mechanisms behind the increased susceptibility of fatty livers to ischemia-reperfusion injury, this review will focus on the available strategies to improve their utilization and will discuss potential future lines of research. 

## 2. Why Are Steatotic Livers More Susceptible to Ischemia-Reperfusion Injury?

Hepatic ischemia-reperfusion injury (IRI) is a sterile inflammatory response commonly encountered during major liver surgery, such as liver resection and liver transplantation, when organ blood supply is restored after a period of ischemia. The pathophysiological bases of IRI have been recently reappraised, identifying mitochondria as the primary targets and initiators of IRI cascade [30,31]. Under ischemic conditions, cell metabolism is switched to anaerobic glycolysis while, at the mitochondrial level, the lack of oxygen interrupts the electron transport chain and causes the accumulation of reduced electron carrier molecules (succinate), initiates reverse electron transfer, and leads to the detachment of flavin mononucleotide from mitochondrial complex I [32]. ATP depletion and lactate accumulation result in electrolyte imbalance and cellular acidosis. When oxygen levels are abruptly restored upon reperfusion, the negative potential across mitochondrial matrix generated during ischemia results in the production of high amounts of reactive oxygen species (ROS) [33]. Hepatocellular ROS initiate the sterile immune response by promoting the release of high mobility group box 1 (HMGB1) and nuclear factor κβ (NF-κβ). HMGB1 and NF-κβ are both central mediators of the reperfusion phase, as their signaling sustains Kupffer cell activation, microcirculation impairment, neutrophil recruitment, and eventually the activation of cell death processes [34,35]. 

It is well known that steatotic livers are extremely vulnerable to IRI but the underlying mechanisms are not completely understood. Evidence from experimental models suggests that the inflammatory response to IRI is different in steatotic and non-steatotic livers [36,37] and that increased mitochondrial oxidative stress and impaired ATP restoration are major determinants of the increased susceptibility of steatotic livers to IRI [38]. 

Mitochondrial uncoupling protein-2 (UCP-2) is a mitochondrial protein that regulates proton leakage across the inner membrane. In steatotic livers, UCP-2 expression is increased to reduce oxidative pressure and ROS production, in an attempt to protect the liver from chronic fat accumulation. However, by diminishing ATP synthesis and reducing ATP baseline levels, UCP-2 overexpression compromises hepatocytes capacity to respond to an acute energy demand, similar to how it occurs during IRI, leading to mitochondrial permeability transition (MPT) and membrane potential collapse [39,40].

In lean livers, cell death due to IRI can occur through different pathways, with apoptosis being the most represented [34,36,41]. However, since apoptosis is an energy-dependent process, the chronic ATP depletion observed in fatty livers may lead to the failure to induce apoptosis in favor of necrosis or other forms of programmed cell death [34,36,42]. Indeed, higher levels of RIPK1 and RIPK3, caspase-9, caspase-1, and iron overload have been observed in fatty degenerated hepatocytes exposed to IRI, suggesting an important role of MPT-driven necrosis, necroptosis, pyroptosis, and ferroptosis, respectively [38]. The considerable overlap and crosstalk between these pathways may have contributed to the confounding and sometimes controversial results reported by the existing studies [42,43,44]. 

Fat droplet accumulation in hepatocytes can cause partial or complete obstruction of sinusoids, resulting in a reduction in sinusoidal blood flow [45,46]. This might be exacerbated, upon graft reperfusion, by the rupture of hepatocyte membrane and the release of fat droplets in the extracellular space, similarly to what happens in lipopeliosis [47,48]. As a result of chronic hypoxic state, steatotic livers are characterized by an increased expression of endothelin (ET-1) and inducible nitric oxide synthase (iNOS). ET-1 and iNOS imbalance aggravates sinusoidal vasoconstriction, worsening microcirculatory damage upon reperfusion [49]. 

The endoplasmic reticulum (ER) serves many roles in the cell including calcium storage, protein synthesis, and lipid metabolism, which are stressed in fatty hepatocytes. Moreover, chaperonin downregulation [50] contributes to ER stress supporting the unfolded protein response (UPR), a signal transduction cascade that ultimately leads to NF-ĸB, JUN N-terminal kinase, and caspase-12 activation [38].

The aforementioned mechanisms, although still partially undisclosed, represent the basis to develop strategies to reduce IRI in fatty livers. 

## 3. Impact of Different Preservation Solutions

Since the introduction of Collins solution in 1969 [51], organ preservation by static cold storage (SCS) has been one of the key elements allowing the expansion of organ transplantation worldwide [52]. The principle of preservation by SCS is slowing down cellular metabolism with hypothermia while preservation solutions prevent or minimize cellular swelling, interstitial edema, intracellular acidosis, and ROS production, and provide energy substrates [52]. Effective preservation is of utmost importance when dealing with steatotic livers. 

First developed in the late 1980s by Belzer and Southard [53], the University of Wisconsin solution (UW) is still considered the gold standard against which other solutions must be compared. UW is a colloid solution with high potassium, low sodium concentration (intracellular solution), and high viscosity due to the presence of hydroxyethyl starch (HES) as an oncotic agent. Histidine–tryptophan–ketoglutarate solution (HTK), which was originally introduced in the 1970s for cardioplegia [54], employs mannitol as an impermeant and does not contain colloids, resulting in decreased viscosity as compared to UW. This solution contains histidine and α-ketoglutarate as energy substrates and buffers, and tryptophan as a membrane stabilizer and antioxidant. Similarly to HTK, Celsior solution (CS) does not contain colloids but it is characterized by high-sodium and low-potassium concentrations (extracellular solution) and was specifically designed to limit calcium overload and ROS production [55]. Institut Georges Lopez-1 solution (IGL-1) is characterized by high-sodium and low-potassium levels (extracellular solution) and by polyethylene glycol (PEG) instead of HES as an oncotic agent, resulting in lower viscosity as compared to UW [56]. UW, HTK, Celsior, and IGL-1 are nowadays the most widely utilized preservation solutions in liver transplantation [52]. In the general population, clinical results with either preservation solution have been shown to be roughly equivalent [57], although large studies based on the European Liver Transplant Registry have shown inferior results with the use of HTK [58,59,60].

In steatotic livers, most studies comparing the efficacy of preservation by different preservation solutions have been conducted in an experimental setting (Table 1).

PEG is a nontoxic, highly soluble neutral polymer capable of preventing edema and cellular membrane destabilization if administered intravenously in a model of warm ischemia-reperfusion injury [61]. The benefits of PEG-containing solutions during cold preservation could be associated with reduced shear stress and improved microcirculation due to reduced viscosity. Indeed, replacing hydroxyethyl starch by PEG results in a much lower viscosity of IGL-1 as compared to UW (1.28 versus 5.7 millipascal-second). Cellular protection is also associated with the reduction of mitochondrial damage by the increased activation of protective cell mechanisms such as adenosine monophosphate-activated protein kinase (AMPK) and endothelial NO synthase (eNOS) [62], as also recently demonstrated in human hepatocytes exposed to IRI in vitro [63].

In 2006, Ben Mosbah et al. first reported the superiority of IGL-1 in preserving steatotic rat livers [64]. Compared to UW, livers preserved with IGL-1 showed less transaminases release, increased bile production, lower malondialdehyde (MDA, a marker of lipid peroxidation and oxidative injury) levels, lower glutamate dehydrogenase (GLDH, a marker of mitochondrial injury) activity, and reduced vascular resistance. The authors postulated that nitric oxide (NO) was involved in the IGL-1 protection against IRI, as suggested by the overexpression of eNOS in the IGL-1 group and by the suppression of IGL-1 protective effects when a NO-inhibitor was added to the preservation solution. The same group then investigated the mechanistic aspects of IGL-1’s apparent superior preservation of steatotic livers in a series of subsequent experiments. IGL-1 enriched with either insulin-like growth factor-1 or epidermal growth factor further increased eNOS activation and improved protection against IRI [65,66]. High levels of hypoxia-inducible factor 1-alpha (HIF-1α) were found in livers preserved with IGL-1, and the overexpression of heme-oxygenase 1 (HO-1), one of the HIF-1α downstream genes, supported the cytoprotective role of this signaling pathway [67]. Trimetazidine, an anti-ischemic drug, enhanced HIF-1α and sirtuin 1 induction and reduced HMGB1 levels, thus promoting autophagy to mitigate IRI [68].

In a study comparing IGL-1 and Celsior, Tabka et al. obtained similar results [69]. Rat livers preserved by IGL-1 showed increased eNOS levels and reduced activation of the pro-apoptotic mitogen-activated protein kinase (MAPK) pathway. Arterial relaxation was found to be highly dependent on NO levels during preservation with IGL-1, corroborating the hypothesis that IGL-1 solution may prevent endothelial dysfunction through eNOS activation. Supplementing IGL-1 with bortezomib, a proteasome inhibitor, resulted in AMPK activation and the downstream expression of eNOS and GSK3β, leading to reduced hepatocellular injury, oxidative stress, and apoptosis [70]. Similarly, carbonic anhydrase II, an enzyme involved in many IRI-related processes, enhanced IGL-1 capacity to induce AMPK and consequently reduce UPR- and MAPK-related events, resulting in superior liver function and histology [71]. Altogether, these results confirmed that IGL-1 benefits in fatty liver preservation are related to AMPK and eNOS activation [72,73]. 

Subsequent studies [56,74,75], besides confirming the advantages of IGL-1 over UW and HTK, showed that the IGL-1 benefits in the preservation of steatotic livers were linked to proteasome inhibition [75], aldehyde dehydrogenase 2 (ALDH2) upregulation [76], and autophagy induction [77]. 

Based on IGL-1 studies, similar improvements were obtained through activation of the AMPK pathway and eNOS induction during SCS using UW. Supplementing UW with trimetazidine, aminoimidazole-4-carboxamide ribonucleoside, carvedilol, or bortezomib during the preservation of steatotic rat livers resulted in lower perfusate transaminases, increased bile production, reduced vascular resistance, and lower MDA and GLDH activity during normothermic reperfusion [78,79,80,81]. 

Eipel et al. [82] investigated the effects of supplementing HTK with erythropoietin for the preservation of steatotic mice livers. Increased oxygen consumption, better preservation of the endothelium, and a slight reduction in AST levels were observed in the treated group after 2 h or normothermic reperfusion. However, UCP-2 expression was not influenced by erythropoietin supplementation and the signaling pathways explaining better preservation in the treatment group were not completely clarified. 

To further reduce ROS production and cell damage, a new IGL solution (IGL-2) was developed with higher PEG (5 versus 1 gr/L) and glutathione concentrations, and the addition of histidine and mannitol instead of raffinose as an impermeant [83]. Importantly, IGL-2 was designed for use during both SCS and machine perfusion, possibly avoiding the need for repeated graft flushing between different phases of organ preservation [84]. In fatty livers, preservation by IGL-2 resulted in reduced mitochondrial injury and oxidative stress, as reflected by increased levels of HO-1, glutathione, ALDH2, and mitochondrial complex I and II, all key actors in the response to IRI [83,84,85]. Interestingly, livers stored with IGL-2 retained the lowest amount of water during preservation, suggesting that PEG could decrease the interstitial formation. 

In conclusion, experimental evidence suggests that PEG-containing solutions provide advantages in terms of mitochondrial integrity and protection against oxidative stress and that IGL-1 and IGL-2 seem the most appropriate preservation solutions for SCS of fatty livers. Theoretically, use of IGL-2 would also be associated with the logistical advantages of using the same solution for SCS and machine perfusion. However, these findings must be interpreted with caution due to the lack of experimental models involving transplantation. Furthermore, IGL-2 is still awaiting approval for clinical use and the advantages of PEG-containing solutions should be confirmed in clinical studies.

**Table 1:** Experimental studies evaluating the impact of different preservation solutions in the preservation of steatotic livers.

Author, Year	Intervention	Experimental Model	Findings
Ben Mosbah et al., 2006 [64]	IGL-1 (vs. UW)	24 h SCS followed by 2 h normothermic reperfusion in Zucker rat livers	Lower perfusate transaminase, MDA, and GLDH levels; improved bile production; lower vascular resistance. Inhibition of NO production suppressed IGL-1 effects.
Ben Mosbah et al., 2007 [78]	UW (+trimetazidine +aminoimidazole-4-carboxamide ribonucleoside)	24 h SCS followed by 2 h normothermic reperfusion in Zucker rat livers	Lower perfusate transaminase, MDA, and GLDH levels; improved bile production; lower vascular resistance. Increased AMPK activation. Inhibition of AMPK suppressed the protective effects.
Ben Mosbah et al., 2010 [79]	UW (+carvedilol)	24 h SCS followed by 2 h normothermic reperfusion in Zucker rat livers	Lower perfusate transaminase, MDA, and GLDH levels; improved bile production; lower vascular resistance; increased ATP. Increased AMPK activation.
Zaouali et al., 2010 [67]	IGL-1 (+trimetazidine)	24 h SCS followed by 2 h normothermic reperfusion in Zucker rat livers	Lower perfusate transaminase, MDA, and GLDH levels; improved bile production; lower vascular resistance. Increased levels of HIF-1α and downstream genes. Better results and HIF-1α induction after addition of trimetazidine. Inhibition of NO production suppressed the protective effects.
Zaouali et al., 2010 [66]	IGL-1 (+IGF-1)	24 h SCS followed by 2 h normothermic reperfusion in Zucker rat livers	Compared to IGL-1 alone: increased NO production, lower perfusate transaminase, MDA, and GLDH levels; improved bile production; lower vascular resistance, reduced oxidative stress.
Zaouali et al., 2010 [65]	IGL-1 (+EGF)	24 h SCS followed by 2 h normothermic reperfusion in Zucker rat livers	Compared to IGL-1 alone: increased NO production, lower perfusate transaminase, MDA, and GLDH levels; improved bile production; lower vascular resistance, reduced oxidative stress; increased ATP.
Eipel et al., 2012 [82]	HTK (+erythropoietin)	24 h SCS followed by 2 h normothermic reperfusion in ob/ob mice livers	Compared to HTK alone: lower perfusate AST; improved endothelial integrity; higher oxygen consumption.
Bejaoui et al., 2014 [70]	IGL-1 (+bortezomib)	24 h SCS followed by 2 h normothermic reperfusion in Zucker rat livers	Compared to IGL-1 alone: activation of AMPK signaling, lower perfusate transaminase; improved bile production; lower vascular resistance, apoptosis inhibition. Inhibition of AMPK expression reduced IGL-1 protective effects.
Zaouali et al., 2013 [80]	UW (+bortezomib)	24 h SCS followed by 2 h normothermic reperfusion in Zucker rat livers	Lower perfusate transaminase, MDA, and GLDH levels; improved bile production; lower vascular resistance. Increased AMPK activation.
Bejaoui et al., 2015 [71]	IGL-1 (+carbonic anhydrase II)	24 h SCS followed by 2 h normothermic reperfusion in Zucker rat livers	Compared to IGL-1 alone: activation of AMPK signaling, lower perfusate transaminase; improved bile production; increased ATP; downregulation of MAPK and UPR pathway; apoptosis inhibition.
Bejaoui et al., 2015 [62]	PEG preconditioning	24 h SCS followed by 2 h normothermic reperfusion in Zucker rat livers	Lower perfusate transaminase, and GLDH levels; lower vascular resistance. Increased AMPK activation.
Tabka et al., 2015 [69]	IGL-1 (vs. Celsior)	24 h SCS followed by 2 h normothermic reperfusion in Sprague-Dawley rats rat livers	Increased NO production, lower perfusate transaminase, MDA, and GLDH levels; improved bile production; lower vascular resistance, reduced oxidative stress, downregulation of MAPK pathway.
Zaouali et al., 2017 [68]	IGL-1 (+trimetazidine)	24 h SCS followed by 2 h normothermic reperfusion in Zucker rat livers	Compared to IGL-1 alone: lower perfusate transaminase and GLDH levels; increased levels of sirtuin 1 and reduced levels of HMGB1 and TNFα.
Zaouali et al., 2017 [75]	IGL-1 (vs. UW)	24 h SCS followed by 2 h normothermic reperfusion in Zucker rat livers	Lower perfusate transaminase and GLDH levels; increased ATP; reduced levels of HMGB1 and TNFα. Proteasome inhibition.
Panisello-Roselló et al., 2017 [56]	IGL-1 (vs. HTK)	24 h SCS of Zucker rat livers	Lower perfusate transaminase and GLDH levels; increased ATP; reduced levels of HMGB1 and TNFα. Proteasome inhibition. Increased AMPK activation.
Panisello-Roselló et al., 2018 [76]	IGL-1 (vs. HTK vs. UW)	24 h SCS of Zucker rat livers	Lower perfusate transaminase levels; increased ATP; reduced apoptosis. ALDH2 upregulation.
Panisello-Roselló et al., 2018 [74]	IGL-1 (vs. HTK)	24 h SCS of Zucker rat livers	Lower perfusate transaminase and GLDH levels; reduced membrane mitochondrial depolarization; reduced apoptosis; reduced levels of HMGB1; increased autophagy.
Lopez et al., 2018 [86]	IGL-1 (vs. HTK vs. IGL-0 *)	24 h SCS of Zucker rat livers	Lower perfusate transaminase levels, preserved glycocalyx integrity.
Bardallo et al., 2021 [83]	IGL-2 (vs. IGL-1, vs. IGL-0 *)	24 h SCS of Zucker rat livers	Lower perfusate transaminase and GLDH levels; increased ATP; increased autophagy; ALDH2 upregulation.
Bardallo et al., 2022 [85]	IGL-2 (vs. IGL-1, vs. IGL-0 *)	24 h SCS of Zucker rat livers	Increased ATP; reduced succinate accumulation; increased complex I and complex II levels: increased HO-1; increase glutathione levels; reduced oxidative stress.
Asong-Fontem et al., 2022 [84]	IGL-2 (vs. UW)	24 h SCS +/− 2 h HOPE followed by 2 h normothermic reperfusion in Zucker rat livers	Lower perfusate AST; preserved glycocalyx integrity; reduced levels of HMGB1; increased weight loss (surrogate of edema formation).

* IGL-0 was IGL-1 solution without polyethilen glycol. Abbreviations: ALDH2, aldehyde dehydrogenase 2; AMPK, adenosine monophosphate-activated protein kinase; EGF, epidermal growth factor; GLDH, glutamate dehydrogenase; HIF-1α, hypoxia-inducible factor 1-alpha; HMGB1, high mobility group box 1; HO-1, heme oxygenase 1; HOPE, hypothermic oxygenated perfusion; MAPK, mitogen-activated protein kinase; MDA, malondialdehyde; NO, nitric oxide; SCS, static cold storage; TNFα, tumor necrosis factor alpha; UPR, unfolded protein response.

## 4. Ischemic Preconditioning

In the setting of LT, ischemic preconditioning (IP) consists in exposing the liver to a brief period of warm ischemia by clamping the hepatic pedicle, followed by a period of reperfusion before SCS. The protective effects of IP against IRI were first described in the heart in the 1980s [87] and were subsequently confirmed in the liver, mostly in warm IRI models or in the setting of liver resection [88,89,90]. IP-activated molecular pathways improve energy storage, pH, and ion homeostasis, and mitochondrial function during the subsequent ischemic period, as well as stimulate antioxidant production and heat shock protein expression. This results in reduced ROS production, inflammatory cytokines release, inflammation, and vasoconstriction upon reperfusion [91]. In clinical LT, pathways activated by IP also explain part of the benefits of normothermic regional perfusion in donation after circulatory determination of death [92,93,94,95,96,97,98,99,100,101].

Serafin et al. were the first to apply IP in fatty livers [102]. Five minutes of ischemia followed by ten minutes of reperfusion prior to prolonged warm ischemia significantly improved the hepatic response to IRI. A consistent number of following preclinical studies confirmed the protective effects of IP in steatotic livers (Table 2). The majority of studies adopted the same model of five minutes of warm ischemia and ten minutes of reperfusion, followed in most cases by 60 min of warm ischemia. IP was associated with improved survival [102,103] and reduced hepatocyte injury, as reflected by lower transaminases levels and decreased histological damage [102,103,104,105,106,107,108,109,110,111,112,113]. In models of SCS followed by transplantation, preconditioned steatotic livers showed lower mortality and improved graft function [72,114,115,116,117]. However, Chu et al. [118] reported that IP was effective with mild steatotic livers but failed to protect against IRI in moderate/severe steatosis, suggesting that other/additional interventions should be considered in livers with >30% steatosis. Moreover, most experimental studies used genetic or diet-induced models of steatosis that do not fully reproduce the characteristics of human hepatic steatosis, which limit the translatability of their findings to the clinical LT setting. 

**Figure 1:**
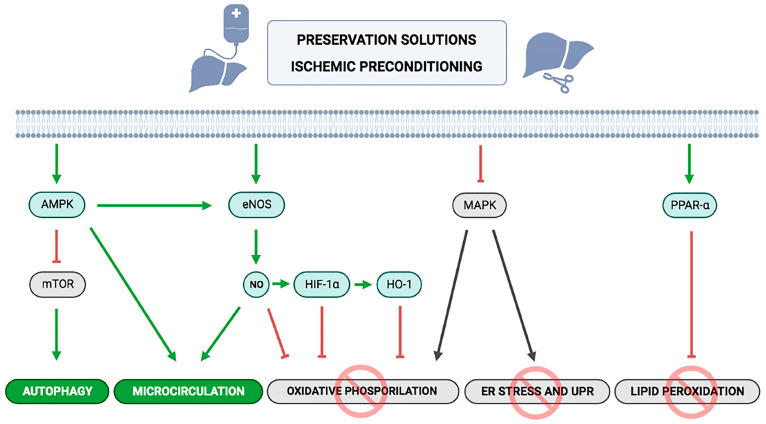
Common protective mechanisms of preservation solutions and ischemic preconditioning.

At the molecular level, it is interesting to note that the cytoprotective mechanisms of IP are similar to those of some preservation solutions (Figure 1) [84,86]. IP enhances AMPK activation and NO production by eNOS [72] and modulates lipid peroxidation by downregulating proliferator-activated receptor-α (PPAR-α) and upregulating proliferator-activated receptor-α [110,114,116]. Casillas-Ramirez et al. [104] showed that inducing PPAR-γ expression by IP and/or angiotensin II inhibitors resulted in superior protection against IRI. Sirtuin 1 activation was associated with enhanced AMPK and eNOS induction, confirming increased NO production after IP [107]. NO is a key factor in the protective effects of IP, being able to attenuate oxidative stress, reduce neutrophil accumulation, and improve microcirculation [72,102,103,106,107,108,109,115,116]. Furthermore, IP reduces MAPK activation and increases HO-1 expression, thus protecting against oxidative stress and IRI progression [109,110], and modulates the balance between proinflammatory and antinflammatory cytokines, reducing IL-1 and IL-6 expression and upregulating IL-10 [103,104,110,112]. 

Despite the promising results from preclinical studies, the application of IP in clinical LT has produced conflicting results. Two prospective randomized studies involving more than 100 major liver resections reported reduced transaminases and bilirubin levels in the IP arm [119,120]. Similar results were observed with marginal grafts subjected to IP prior to liver transplantation [121,122,123,124]. By contrast, another prospective randomized trial involving 101 liver transplants, including 56 steatotic grafts, showed opposite results, with increased transaminase levels in the IP group [125]. In 2008, a Cochrane review did not show evidence to support or refute the use of IP in the liver transplantation setting [125], while a more recent meta-analysis revealed some beneficial effect of IP concerning postoperative AST levels and mortality rates [126]. These controversial results probably contributed to the gradual fading of interest in IP, with the last randomized trial published in 2009. Moreover, as none of the existing clinical studies were specifically designed to investigate the effects of IRI on steatosis, a definitive conclusion with regards of its benefits in this setting cannot be drawn. 

**Table 2:** Preclinical studies on IP in steatotic livers.

Author, Year	Animal	Model	Protocol	Ischemia	Findings
Serafin et al., 2002 [102]	Rat	Partial IRI	5 min + 10 min 10 min + 10 min 10 min + 15 min	60 min, warm	5 + 10 min IP protocol produced better results. Increased survival; reduced ALT; reduced necrosis; lower MDA; increased GSH; increased blood flow. Inhibition of NO production suppressed the protective effects.
Selzner et al., 2003 [113]	Mouse	Partial IRI	10 min + 10 min	75 min, warm	Reduced AST; reduced necrosis and apoptosis; increased ATP.
Serafin et al., 2004 [103]	Rat	Partial IRI	5 min + 10 min	60 min, warm	Increased survival; reduced ALT; reduced necrosis; lower MDA; reduced IL-1b and increased IL-10 Inhibition of NO production suppressed the protective effects.
Fernandez et al., 2004 [115]	Rat	LT	5 min + 10 min	6 h, cold	Reduced AST and ALT; reduced necrosis; reduced MPO; modulation of ROS-generating system and lipid peroxidation. Inhibition of NO production suppressed the protective effects.
Carrasco-Chaumel et al., 2005 [72]	Rat	LT	5 min + 10 min	6 h, cold	Reduced AST and ALT; reduced necrosis, increased NO production; activation of AMPK signaling. Inhibition of NO production suppressed the protective effects.
Niemann et al., 2005 [117]	Rat	LT	10 min + 10 min	4 h, cold	Increased survival; increased ATP; lower lactate
Koti et al., 2005 [108]	Rat	Partial IRI	5 min + 10 min	45 min, warm	Reduced AST and ALT; increased ATP; increased oxygenation and microcirculation
Massip-Salcedo et al., 2006 [109]	Rat	Partial IRI	5 min + 10 min	60 min, warm	Reduced AST and ALT; reduced necrosis; increased HO-1; downregulation of MAPK pathway. Inhibition of NO production and/or HO-1 suppressed the protective effects.
Saidi et al., 2007 [112]	Rat	Partial IRI	10 min + 15 min	75 min, warm	Reduced AST; reduced IL-6; reduced necrosis.
Massip-Salcedo et al., 2008 [110]	Rat	Partial IRI	5 min + 10 min	60 min, warm	Reduced ALT; reduced necrosis; lower MDA; reduced IL-1b; PPAR-α upregulation; adiponectin downregulation; downregulation of MAPK pathway. Inhibition of PPAR-α suppressed the protective effects.
Casillas-Ramirez et al., 2008 [104]	Rat	Partial IRI	5 min + 10 min	60 min, warm	Reduced ALT; reduced IL-1, reduced necrosis; reduced angiotensin II. ACE-inhibitors produced same benefits.
Rolo et al., 2009 [111]	Rat	Partial IRI	5 min + 10 min	90 min, warm	Reduced AST and ALT; reduced membrane mitochondrial depolarization; increased ATP; reduced MPT induction
Hafez et al., 2010 [105]	Rabbit	Partial IRI	5 min + 10 min	60 min, warm	Reduced AST and ALT; increased oxygenation and microcirculation, improved bile quality
Casillas-Ramirez et al., 2011 [114]	Rat	LT	5 min + 10 min	6 h, cold	Reduced AST and ALT; reduced necrosis. Increased AMPK activation; PPAR-γ downregulation. Inhibition of AMPK suppressed the protective effects.
Jiang et al., 2013 [106]	Rat	Partial IRI	5 min + 10 min 8 min + 10 min 10 min + 10 min 15 min + 10 min	30 min, warm	5 + 10 min and 8 + 10 min IP protocols produced better results. Reduced AST, ALT and LDH; increased NO production, reduced MPO; lower MDA;
Pantazi et al., 2014 [107]	Rat	Partial IRI	5 min + 10 min	60 min, warm	Reduced AST; reduced necrosis and apoptosis; increased NO production; activation of AMPK signaling. Increased levels of sirtuin 1. Inhibition of sirtuin 1 suppressed the protective effects.
Chu et al., 2015 [118]	Rat	SCS	10 min + 10 min	24 h, cold	Reduced complex I injury. Protective effects only with mild steatosis, not with moderate/severe steatosis.
Jimenez-Castro et al., 2015 [116]	Rat	LT	5 min + 10 min	6 h, cold	Increased survival; reduced ALT and AST; increased NO production, reduced MPO; lower MDA; PPAR-α upregulation; PPAR-γ downregulation. Inhibition of NO production suppressed the protective effects.

Abbreviations: AMPK, adenosine monophosphate-activated protein kinase; GSH, glutathione; HO-1, heme oxygenase 1; IP, ischemic preconditioning; LT, liver transplantation; MDA, malondialdehyde; MPO, myeloperoxidase; MPT, mitochondrial permeability transition; PPAR-α, proliferator-activated receptor-α; PPAR-γ, proliferator-activated receptor-γ.

## 5. Hypothermic Oxygenated Machine Perfusion

The modern era of clinical liver machine perfusion began in 2010 with the publication by Guarrera et al. [127] on the first clinical series of livers treated with hypothermic machine perfusion. Compared to patients transplanted with livers preserved by SCS using UW solution, recipients of livers treated with end-ischemic hypothermic machine perfusion showed reduced postoperative markers of IRI (AST, ALT, bilirubin, and creatinine peak levels) and shorter postoperative stay. The positive impact of this approach on LT outcome has been subsequently confirmed in several clinical studies [101,128,129,130,131,132,133,134,135,136,137,138,139,140,141,142,143] and four randomized controlled trials [144,145,146,147]. As most groups apply hypothermic machine perfusion with active perfusate oxygenation, this technique in frequently referred to as hypothermic oxygenated machine perfusion (HOPE). The clinical benefits of HOPE include a lower rate of postreperfusion syndrome, acute kidney injury, and postoperative complications, as well as a reduced incidence of early allograft loss and ischemic cholangiopathy [137,139,144,145,146,147,148]. Although HOPE benefits have been linked to the continuous perfusion and washout of metabolic waste products, the delivery of oxygen at low temperatures represents the key element explaining its efficacy. By allowing tissue reoxygenation, HOPE contrasts the noxious metabolic effects of ischemia at their roots, preventing mitochondrial respiratory chain dysfunction that results in reverse electron transfer and ROS production upon graft reperfusion [149,150,151,152,153]. During HOPE, the mitochondria are reprogrammed to a fully oxidized state, favoring succinate metabolism and preventing its accumulation [152,153]. As a result, ROS production and subsequent local and systemic inflammation are reduced when the graft is reperfused into the recipients, which translate into improved postoperative graft function and clinical outcomes.

Additionally, HOPE offers an opportunity to assess graft damage and function before LT, which is of particular interest when fatty liver grafts are considered for LT. Since the early clinical series, a correlation between transaminase level in HOPE perfusate and their levels in the recipient after transplantation have been observed [127,134,154]. A study from our group [155] confirmed these findings, showing that perfusate parameters, especially ALT, are associated with the development of early allograft dysfunction [10] after LT. The Zurich group, which is one of the pioneering groups in machine perfusion and has thoroughly studied different aspects of HOPE, identified mitochondrial complex I cofactor flavin mononucleotide (FMN) as a promising marker of liver viability during HOPE, closely correlating with clinical outcomes and potentially driving graft acceptance and allocation [152,156]. 

Given its mechanism of action, HOPE represents a promising strategy in reducing IRI in fatty livers, which are particularly exposed to oxidative stress and exhibit higher ROS production upon reperfusion. In an isolated perfused rat liver model, Bessems et al. [157] showed that, by replacing 24 h SCS with continuous HOPE, liver grafts with 30–60% steatosis showed reduced cytolysis, better hyaluronic acid clearance, increased bile and urea production, higher oxygen consumption and ATP levels, and preserved tissue morphology, with reduced necrosis and edema formation.

In a subsequent study, Kron et al. [135] transplanted rat liver grafts with severe (≥60%) macrosteatosis after 12 h of SCS alone, or after 12 h SCS followed by 1 h HOPE. The treated livers showed a marked decrease in hepatocellular injury, oxidative stress, HMBG1 release, and endothelial cell activation, as well as improved microcirculation and replenished energy stores. Animal survival was higher in the HOPE group and was comparable to that of rats transplanted with lean livers. Similarly to other studies from the same group [151], the authors observed that perfusing livers with the same perfusate deoxygenated using nitrogen gas suppressed HOPE protection, confirming the fundamental role of tissue oxygenation during HOPE. Similar protective effects were reported by Asong-Fontem et al. [84] by using IGL-2 solution for both SCS and HOPE. 

**Figure 2:**
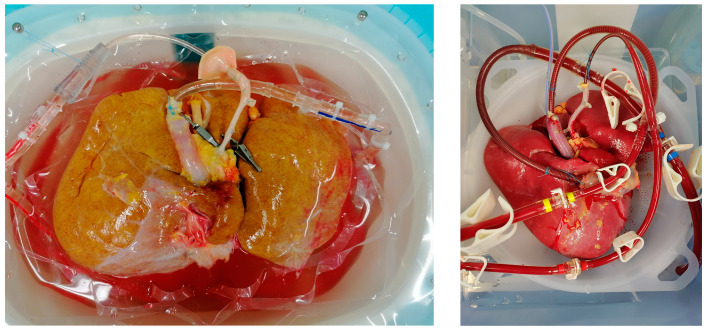
Steatotic liver grafts undergoing D-HOPE and NMP.

Clinical data on HOPE in fatty livers preservation are still limited. The D-HOPE DCD trial [147], which has been the first randomized controlled trial (RCT) on dual (i.e., perfusion of both portal vein and hepatic artery) HOPE (Figure 2), was focused on grafts from donors after circulatory death (DCD), with the primary endpoint being the 6-month incidence of non-anastomotic strictures requiring treatment. In this trial, four LT were cancelled after randomization, in two cases due to “massive” steatosis, and no information was provided concerning the degree of steatosis in the included grafts. Presumably, inclusion of livers with moderate or severe steatosis was avoided to prevent overlap of different risk factors in the same donor. Three subsequent RCTs explored the value of HOPE in livers from extended criteria donors after brain death (DBD). In the study by Czigany et al. [144], steatosis ≥ 40% was one of the inclusion criteria, but only two (9%) of donors in the HOPE group had > 30% MaS and specific outcomes were not provided. In the RCT by Ravaioli et al. [145], 25 initially randomized livers were discarded due to an unacceptable risk after macroscopic (*n* = 10) or microscopic (*n* = 11) assessment and median MaS was 2% in the HOPE group. More recently, Schlegel et al. [146] reported the results of a multicenter trial involving 10 transplant centers in Europe evaluating incidence and gravity of surgical complication at 1 year, but data on graft steatosis were not reported. 

As of today, data on HOPE for fatty liver grafts are limited to few cases gathered from retrospective studies including a mixture of donors matching the definition of “extended criteria” for different reasons (Table 3) [137,138,139,141,155,158]. In the same aforementioned paper, Kron et al. [135] reported the results of the HOPE application in six human livers (DCD, *n* = 5) with 20–40% macrosteatosis and 20–90% macrosteatosis, which were successfully transplanted after a median time of 2.3 h of HOPE. When compared to a matched group of 12 LT performed with steatotic grafts preserved by SCS (DBD, *n* = 12), recipients of HOPE-treated livers had lower AST peak, lower need for renal replacement therapy, shorter ICU stay, and better 1-year survival. Notably, 25% of patients in the SCS group developed primary non-function, 25% underwent re-transplantation, 75% needed hemodialysis, and only 42% were alive after 1 year. The experience of our group with HOPE for liver grafts with moderate or severe MaS has been less clear cut. After an initial favorable experience [138], the rare cases of primary non-function or severe graft dysfunction in HOPE-treated livers have been almost invariably observed in steatotic grafts [137].

As originally reported by Guarrera et al. [134], these livers with significant MaS are particularly stiff and require higher perfusion pressures during HOPE, which also in our experience represents a negative prognostic sign with regards to postoperative graft function. Furthermore, our study on the predictive value of perfusate parameters during HOPE showed that MaS was the only independent predictor of EAD [155]. 

Overall, available evidence suggests that, while brilliant results can be achieved with HOPE-treated fatty livers, this technique may occasionally be insufficient to recondition these already severely damaged grafts, which leads to the necessity of assessing their viability before LT [152,156,158,159,160,161,162]. An interesting treatment algorithm has recently been proposed by the Zurich group, which has developed a machine allowing normothermic perfusion for several days [163,164]. Based on FMN perfusate levels during HOPE, severely damaged grafts (including those with ≥30 MaS) would be either discarded or treated by long-term normothermic machine perfusion (NMP) to test their viability and assess whether they are suitable for LT [165]. 

## 6. Subnormothermic Machine Perfusion

Subnormothermic machine perfusion (SNMP) is a dynamic preservation technique characterized by perfusion at 20 °C. Vairetti et al. [166] first investigated the use of SNMP to preserve steatotic rat livers using an acellular perfusate. SNMP was compared to SCS and HMP in lean and steatotic livers during a 2 h reperfusion at 37 °C. Livers undergoing SNMP showed improved cytolytic enzyme release, bile production, glycogen stores, ATP replenishment, and oxidative stress compared to both SCS and HMP groups. A following study by the same group used an equivalent model to demonstrate reduced hepatocyte and sinusoidal apoptosis in fatty livers preserved with SNMP, resulting in preserved hepatic ultrastructure and improved microcirculation [167]. With respect to the biliary tree, SNMP has been associated with the improved preservation of bile canaliculi, as demonstrated by increased dipeptidylpeptidase-IV activity and expression, used as a marker of biliary tree morphology [168]. Similarly, Okamura et al. [169] showed that, compared to SCS, SNMP-preserved steatotic livers released less ALT and mitochondrial glutamate dehydrogenase during reperfusion at normothermic temperature. Bile production, ATP levels, lipid peroxidation, and tissue glutathione were all significantly improved by SNMP, while electron microscopy revealed a reduction in sinusoidal microvasculature and hepatocellular mitochondria injury.

In a preliminary study on discarded human livers undergoing SNMP for 3 h, impaired NO pathway activation was observed in steatotic livers compared to non-steatotic livers, confirming the crucial role of NO in preventing endothelial dysfunction [170]. A recent metabolomic analysis compared the preservation of discarded steatotic human livers at either subnormothermic or normothermic temperatures [171]. Although higher ATP regeneration was observed during SNMP, this was associated with a concomitant glutathione depletion. Authors concluded that the impaired antioxidant capacity associated with SNMP may worsen IRI and warrants caution in translating this technique to clinical practice. As of today, SNMP has not been evaluated as a standalone technique in a clinical trial. 

An interesting concept, first prosed by Minor et al. [172], is that of controlled oxygenated rewarming (COR), which consists in progressively rewarming the liver under active oxygenation, before reperfusion at 37 °C. In a DBD pig model, this group observed that COR compared favorably with SCS, HMP, and SNMP in terms of hepatocellular damage, ROS production, expression of inflammatory mediators, and portal vein flow during subsequent normothermic reperfusion. This concept was clinically implemented by the Groningen group in the DHOPE-COR-NMP trial, which demonstrated the effectiveness of the sequential application of D-HOPE and NMP, separated by a brief period of COR, in recovering a substantial number of livers for LT which had been initially discarded [173,174]. This approach combines the beneficial effects of D-HOPE on the mitochondrial respiratory chain with the possibility of testing viability during NMP. However, data concerning the application of this protocol in steatotic livers are lacking.

**Table 3:** Clinical MP studies on livers with macrovesicular steatosis ≥ 30%.

Author, Year	n	Intervention	Findings
Guarrera et al., 2015 [134]	1	End-ischemic HMP	A patient receiving a DBD graft with 40–50% MaS developed PNF. High-portal pressure and elevated effluent transaminases were observed during HMP.
Kron et al., 2017 [135]	6	End-ischemic HOPE	As compared to SCS, recipients of HOPE-treated livers (DCD, n = 5) had lower transaminase peak, lower dialysis requirement, shorter ICU stay, and better survival.
Rayar et al., 2021 [141]	1	End-ischemic D-HOPE	One patient receiving a graft with 30% steatosis had good function after LT and was alive with a functioning graft at 3-month follow-up.
Patrono et al., 2020 [155]	5	End-ischemic D-HOPE	Graft MaS-influenced levels of perfusate AST, ALT, LDH, glucose, lactate, and pH and predicted development of EAD after LT. Of 5 recipients of livers with MaS ≥ 30%, one required re-LT.
Patrono et al., 2022 [137]	12	End-ischemic D-HOPE	Of 12 recipients of livers with ≥30% MaS 5 has AST peak > 6000, 50% developed grade 2–3 AKI, 2 (16.7%) developed EAF, and 1 (8.3%) died.
Watson et al., 2018 [175]	1	End-ischemic NMP	One liver described as “very steatotic” accepted for research and not transplanted. Perfusate ALT at 2 h = 7542 IU/L; no glucose metabolism.
Ceresa et al., 2019 [176]	1	End-ischemic NMP	Of three (9.7%) discarded livers, one DBD liver with 80% MaS was discarded due to insufficient lactate clearance and lack of bile production and glucose metabolism.
Mergental et al., 2020 [177]	2	End-ischemic NMP	Of 9 (29%) discarded livers, 2 had moderate or severe MaS. Prevalence of medium-large droplet steatosis was higher among discarded livers (77.8% vs. 40.9%). No liver with MaS ≥ 30% was accepted for LT.
Fodor et al., 2021 [178]	3	End-ischemic NMP	Of 59 included patients, 3 (5.1%) received a liver with MaS ≥ 30%. Specific outcomes were not reported.
Patrono et al., 2022 [179]	14	End-ischemic NMP	Of 14 evaluated livers, 10 (71%) were transplanted but 2 (14%) developed PNF, whereas post-LT graft function was good in the remaining patients
He et al., 2018 [180]	1	IFLT	A DBD liver with 85–95% MaS was procured, preserved and successfully transplanted by IFLT.
Chen et al., 2021 [181]	26	IFLT	A total of 26 livers with moderate (n = 16) or severe (n = 10) MaS were included, of which 6 were treated by IFLT. IFLT was associated with reduced AST, GGT, and creatinine peak after LT, and lower EAD rate (0% versus 60%, *p* = 0.001).

Abbreviations: HMP, hypothermic machine perfusion; DBD, donation after brain death; MaS, macrovesicular steatosis; PNF, primary non-function; HOPE, hypothermic oxygenated machine perfusion; SCS, static cold storage; DCD, donation after circulatory determination of death; ICU, intensive care unit; D-HOPE, dual hypothermic oxygenated machine perfusion; LT, liver transplantation; AKI, acute kidney injury; EAF, early allograft failure; NMP, normothermic machine perfusion; IFLT, ischemia-free liver transplantation.

## 7. Normothermic Machine Perfusion

In contrast with HOPE and SNMP, normothermic machine perfusion aims to reproduce a physiological environment in which the liver is supplied with oxygen and nutrients at 37 °C [182,183]. While NMP was initially meant to substitute most of the SCS time [184] (normothermic machine preservation), it is nowadays most frequently applied after an initial period of cold preservation (end-ischemic or “back-to-base” approach), with the main objective of assessing liver viability [176,177,178,182,183,185,186,187]. Besides allowing the restoration of liver metabolism and cellular ATP content, NMP results in the modulation of apoptosis and immune response and enhancement of regenerative pathways [188,189,190,191,192,193]. Furthermore, NMP may be used as a platform to administer organ therapeutics [194,195,196,197,198]. 

Three RCTs have investigated the clinical advantages of NMP over SCS. In the pivotal COPE trial, Nasralla et al. [199] demonstrated that upfront NMP initiated at the donor hospital is associated with reduced post-LT AST peak, which was achieved despite a 50% lower discard rate and longer preservation time. In this study, NMP also resulted in a lower incidence of postreperfusion syndrome and EAD. However, quantification of graft steatosis was based on the macroscopic assessment by the retrieving surgeon, limiting the available information about the effectiveness of NMP in this setting. The PROTECT trial [200] confirmed that upfront NMP is associated with a lower incidence of EAD, as well as reduced histological signs of IRI and a lower incidence of ischemic-type biliary lesions at 6- and 12-month follow-up. By study design, however, only livers with ≤40% MaS were included, and the degree of steatosis for the included livers was not clearly reported. The trial by Ghinolfi et al. [201] investigated the benefits of end-ischemic NMP in elderly DBD donors and showed that, while NMP did not result in significantly improved clinical outcomes, it was associated with superior preservation as assessed by electron microscopy. No graft with MaS ≥ 30% was included in this study. 

Data on the clinical effectiveness of NMP in grafts with moderate or severe steatosis are scarce. When NMP has been used with a back-to-base approach, graft steatosis has been a frequent reason leading to organ discard a priori or due to failure to meet viability criteria (Table 3). Watson et al. [175] reported on a liver described as “very steatotic” which was accepted for research and not transplanted. In the study by Ceresa et al. [176] comparing upfront versus back-to-base groups, three (9.7%) grafts (DCD, *n* = 2; DBD, *n* = 1) were discarded in the back-to-base group. Among these, the DBD graft showed severe (80%) MaS and was discarded due to insufficient lactate clearance, as well as lack of bile production and glucose metabolism. In the VITTAL trial from the Birmingham group [177], the prevalence of large–medium droplet steatosis > 30% was 77.8% among discarded organs versus 40.9% in those that were accepted. However, no accepted graft had moderate or severe MaS at centralized histological evaluation, while among nine discarded grafts, two had moderated or severe MaS. Another intriguing finding from this study was the discrepancy in MaS quantification between pathologists at the procurement hospital and pathologists at the transplant center. In the series from the Innsbruck group [178], 3 out of the 59 included patients received a graft with MaS ≥ 30%, but specific outcomes were not reported. In a multicenter study including data from our group [179], we investigated the utilization rate and outcomes of livers with MaS ≥ 30% treated by end-ischemic NMP. Of the 14 evaluated livers, 10 (71%) were transplanted, of which 8 (57%) showed good function postoperatively, whereas 2 (14%) developed PNF. This study highlights the difficulties in assessing viability of moderately or severely steatotic livers using the current criteria. 

An extension of the concept of upfront NMP, ischemia-free LT (IFLT) is a procedure by which liver cold ischemia is completely avoided [180]. During IFLT, the liver is cannulated in situ in the donor through portal vein and hepatic artery collaterals. When donor circulation is interrupted, NMP is simultaneously started. In the recipient, vascular anastomosis is performed under continuous NMP, which is ultimately stopped immediately before reperfusing the graft with recipient blood. Interestingly, the first case of human IFLT was reported by the Guangzhou group in a 25-year-old DBD whose liver showed 85–95% MaS. The recipient was a 51-year-old patient suffering from HCC and did not develop postreperfusion syndrome at graft reperfusion, nor any vascular, biliary, or infectious complications. IFLT has been associated with improved post-LT graft function and reduced histological signs of IRI [202]. Recently, the results of the first RCT evaluating IFLT versus conventional LT [203] have shown that IFLT is associated with a significant reduction in EAD rate (6% versus 24%, *p* = 0.044), improved liver function tests (lower transaminase peak and total bilirubin level on postoperative day 7th), a reduction in postreperfusion syndrome, shorter ICU stay, and a lower incidence of ischemic cholangiopathy (8% versus 36%). In a population of young DBD donors (median age = 38 years), the same group compared the outcomes of six grafts with moderate or severe MaS treated with IFLT with those of 20 equally steatotic livers preserved by conventional SCS [181]. Lower peak AST, GGT, and creatinine levels after LT were observed in the IFLT group, as well as a significantly lower rate of EAD (0% versus 60%, *p* = 0.01). Furthermore, no case of PNF or acute kidney injury was observed after IFLT. 

NMP represents an ideal platform for organ-specific pharmacologic interventions. In the setting of steatosis, one intriguing possibility is manipulating lipid metabolism to reduce fatty liver content and perform a so-called “defatting” protocol. Nagrath et al. [204] successfully delivered a defatting cocktail to fatty rat livers using NMP, reducing intracellular lipid content by more than 50%. In a porcine model of mild hepatic steatosis, NMP alone determined a reduction in hepatic fat content from 30% to 15% and fatty livers exhibited perfusate homeostasis, hemodynamics, and bile production comparable to healthy livers [205]. In contrast, the NMP of discarded human livers failed to reduce steatosis even after 24 h of perfusion, contradicting the previous evidence from rodent and pig models [206]. In a study involving 10 discarded human livers randomly assigned to either NMP alone or defatting NMP, the application of a defatting protocol resulted in a 40% decrease in tissue triglyceride content and macrovesicular steatosis after 6 h of perfusion, along with improved mitochondrial function and a reduction in oxidative injury markers and inflammatory cytokines [207]. Notably, all livers treated with the defatting protocol finally met transplant viability criteria, as confirmed by enhanced hemodynamics, lactate clearance, and biliary function. However, as of today no liver treated by a defatting protocol has been transplanted and toxicity of some compounds of the defatting cocktail may limit the application of this approach on the clinical setting. Despite being complicated by important technical hurdles [208,209], long-term NMP might be necessary to allow for effective defatting and the comprehensive viability assessment of fatty liver grafts [163,164,165].

## 8. Conclusions

**Figure 3:**
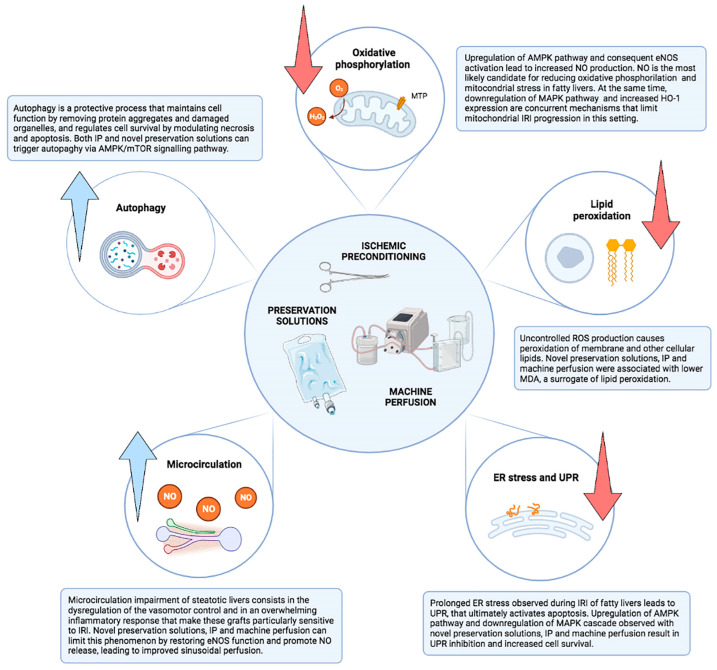
Potential targets to improve preservation of steatotic livers.

After decades of research on the subject (Figure 3), it appears that the definitive strategy to achieve optimal preservation of livers with significant steatosis remains to be found. Despite the encouraging preclinical data, novel preservation solutions and ischemic preconditioning have so far failed to make a difference in the clinical setting. Although dynamic preservation techniques have the potential to improve the preservation of fatty liver grafts and allow for their safe utilization, the available data are still preliminary and must be confirmed in larger clinical trials. Uniform and reproducible steatosis assessment, as well as strict adjustment for potential donor and recipient confounders, will be critical factors in the design of these studies.

## Data Availability

No new data were created or analyzed in this study. Data sharing is not applicable to this article.
